# Traumatic Pseudoaneurysm of Axillary Artery Combined with Brachial Plexus Injury

**DOI:** 10.1371/journal.pone.0113099

**Published:** 2014-11-20

**Authors:** Lin Chen, Feng Peng, Tao Wang, Desong Chen, Jianyun Yang

**Affiliations:** 1 Department of Hand Surgery, Huashan Hospital, Fudan University, Shanghai, China; 2 Key Laboratory of Hand Reconstruction, Ministry of Health, Shanghai, China; 3 Key Laboratory of Peripheral Nerve and Microsurgery, Shanghai, China; University of Washington, United States of America

## Abstract

Traumatic pseudoaneurysm of the axillary artery combined with brachial plexus injury is extremely rare. The factors that influence the symptoms and functional recovery related to this condition are unclear. Nine patients who had sustained this trauma were surgically treated at our unit between June 1999 and November 2010. The cause of trauma, symptoms, signs and examinations of neurological and vascular deficits, and the surgical findings of the involved nerves and vessels were recorded in detail. The functional recovery of vessels and nerves, as well as the extent of pain, were evaluated, respectively. The average length of patient follow-up was 4.5 years (range, 24 months to 11.3 years). After vessel repair, whether by endovascular or operative treatment, the distending, constant, and pulsating pain was relieved in all patients. Furthermore, examination of the radial artery pulse on the repaired side appeared normal at last follow-up. All patients showed satisfactory sensory recovery, with motor recovery rated as good in five patients and fair in four patients. The symptom characteristics varied with the location of the damage to the axillary artery. Ultrasound examination and computed tomography angiography are useful to evaluate vascular injury and provide valuable information for operative planning. Surgical exploration is an effective therapy with results related to the nerve injury condition of the brachial plexus.

## Introduction

Traumatic pseudoaneurysm of the axillary artery combined with brachial plexus injury is extremely rare and has been reported in only a few cases [1], [2], [3]. Traumatic lesions of the axillary artery itself are limited to 2.9% to 9% of major arterial injuries [4]. Traumatic pseudoaneurysms of the axillary artery occasionally occur concomitantly with injury of the brachial plexus, with an incidence ranging from 27% to 44% [5].

Because previous reports on this condition have been limited to case reports, factors that influence the symptoms and functional recovery related to the condition remain unclear. For example, both endovascular repair and operative treatment of axillary artery pseudoaneurysm have been described in different reports [1], [2], [3], [5], [6], [7], but indications for choice of treatment and records of the extent of functional recovery, especially nerve function recovery, have been lacking.

The aims of this study were to assess the cause of trauma, treatment type, and treatment outcomes in a series of patients who had undergone surgery from traumatic pseudoaneurysm of the axillary artery combined with brachial plexus injury.

## Materials and Methods

### Ethics Statement

This research has been approved by the Huashan Hospital Institutional Review Board.

Because all patient's data (including the follow-up data) had been collected before we decided to start this study, and this retrospective data analytical research didn't influence the treatment and prognosis of the participants, the written informed consent from participants was exempted by the Institutional Review Board.

### Patient Data and Surgical Technique

From June 1999 to November 2010, nine patients (six male and three female) who had sustained a pseudoaneurysm of the axillary artery combined with brachial plexus injury were treated in our department. The patients had an average age of 28.7 years (range, 9–45 years). Eight patients sustained injuries to the left upper limb and one patient to the right. Injury was caused by penetrating trauma in four patients and by blunt trauma in the other five. And in the five blunt trauma patients, four suffered injury from a traffic accident and the other one from a fall accident. All had sustained different kinds of neurologic deficits in the injured upper extremities (Table 1). Seven patients were referred to our department from primary hospitals with only a diagnosis of brachial plexus injury without pseudoaneurysm. Two patients were diagnosed with pseudoaneurysms and treated with a vascular stent-graft by a vascular surgeon in the other hospital, and were then referred to our department for treatment of their brachial plexus injuries. The interval from trauma to neurological dysfunction ranged from 0–60 days (mean, 9.4 days). The symptomatic nerve compression duration (interval between the onset of neurological worsening and the day of surgery) ranged between 5 and 120 days (mean, 38.7 days).

**Table 1:** Patient clinicopathological and demographic data.

Case no.	Gender	Age (year)	Lateral side	Cause of injury	Interval from trauma to neurological dysfunction (days)	Duration of nerve compression (days)	Interval from injury to vessel repair (days)	Interval from vessel repair to nerve surgery (days)	Follow-up time (months)
1	F	45	L	A fall	60	30	90	At the same time (0)	63
2	M	16	L	A knife stabbing	9	14	23	At the same time (0)	135
3	F	18	L	A traffic accident	13	21	34	At the same time (0)	41
4	M	26	L	A knife stabbing	At the same time (0)	71	71	At the same time (0)	110
5	M	9	L	A traffic accident	At the same time (0)	5	5	At the same time (0)	30
6	M	40	L	A traffic accident	At the same time (0)	16	16	At the same time (0)	25
7	F	45	L	A glass stabbing	3	19	22	At the same time (0)	27
8	M	26	R	A traffic accident	At the same time (0)	120	84	36	30
9	M	33	L	A knife stabbing	At the same time (0)	52	16	36	24
Average	-	28.7	-	-	9.4	38.7	40.1		53.9

**Figure 1:**
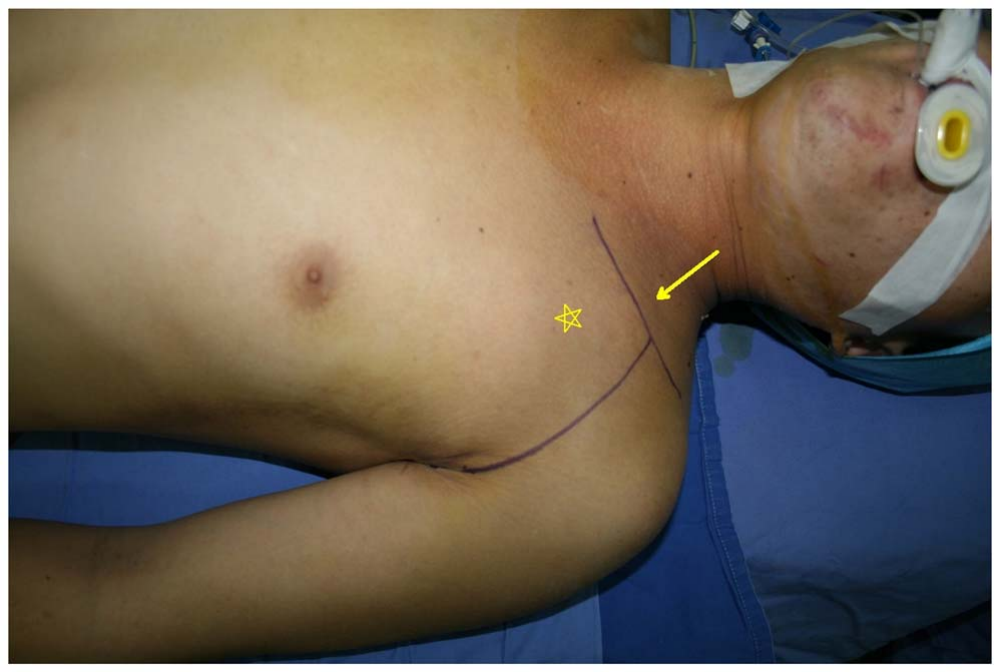
The thrilling mass and the incision in patient no. 6. Patient no. 6 had an expanding and thrilling mass at infraclavicular region. And the incision for the initial exploration was designed as supra- combined with infra-clavicular incision with clavicle fracture fixation simultaneously. (Pentacle: expanding mass; Arrow: supra- combined with infra-clavicular incision).

**Figure 2:**
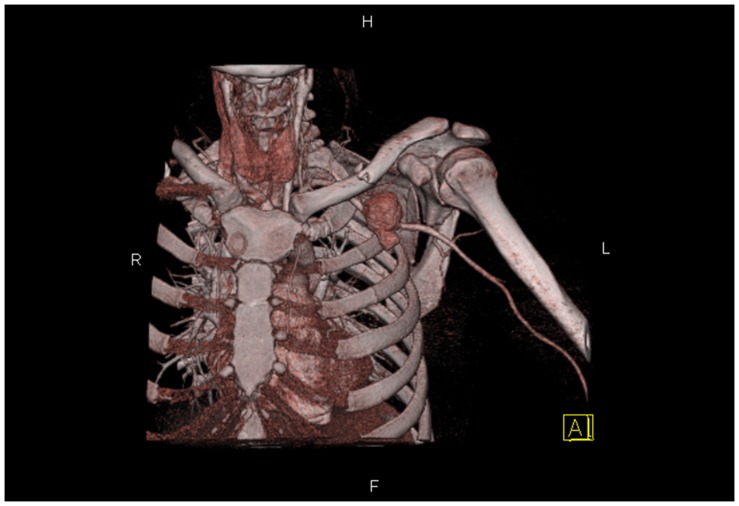
The result of CTA for patient no. 6. Computed Tomographic Angiography (CTA) of patient no. 6 showed the rupture was in the anterior wall of axillary artery.

Two patients had a clear thrill mass in their axillary fossa or infraclavicular region (Figure 1). Four patients were found to have a weak or absent radial artery pulse. Adjunctive evaluations found four patients with bone fractures and six cases with anemia (Table 2). All nine patients underwent Doppler ultrasound examinations. Three patients underwent Computed Tomographic Angiography (CTA; Figure 2) and three underwent Digital Subtraction Angiography (DSA; Table 2).

**Table 2:** The results of clinical and diagnostic examinations.

	Thrill	Radial				Workup	
Case no.	mass	pulse	Concomitance fracture	Hemachrome	Ultrasound*	CTA	DSA
				(g/L)	(cm)		
1	-	+	-	60	20×15	/	/
2	+	-	-	128	7×5	/	
3	±	±	Greater tuberosity fracture	112	7×4.3	/	Laceration at posterior wall of AA
4	±	+	-	125	15×10	/	/
5	±	-	Proximal humeral shaft	80	3×3.5	/	/
			fracture				
6	+	+	Clavicular fracture	90	3.5×2.2	Laceration at anterior wall of AA**	/
7	±	±	-	119	3×2	Laceration at posterior wall of AA	/
8	-	+	Clavicular fracture	121	7×6	/	Laceration at anterior wall of AA
9	-	+	-	79	3.5×2.7	Laceration at inferior wall of AA	Laceration at inferior wall of AA

*: the size of pseudoaneurysm detected by ultrasound.

**: AA  =  axillary artery.

Patients' pain was evaluated using a visual analogue scale (VAS). The characteristics of the pain and its influence on daily living were also recorded (Table 3).

**Table 3:** Pain evaluation scores.

			VAS		
Case no.	Initial	On admission to hospital	The first day after vessel repair	On discharge from our hospital	Final follow-up
1	0	6	2	2	0
2	0	5	3	2	0
3	2	6	3	2	0
4	2	5	2	1	0
5	4	8	2	1	0
6	3	6	2	1	0
7	0	5	2	2	0
8	2	5	3	3	0
9	1	7	3	3	1
Average	1.6	5.9	2.4	1.9	0.1

Patients were grouped by procedure type: in Group 1 (patient nos. 1–7), the pseudoaneurysm and brachial plexus injury were treated as part of the initial surgical exploration; and in Group 2 (patient nos. 8 and 9), a vascular stent-graft was inserted for the pseudoaneurysm before the surgery for the brachial plexus injury.

### Surgical Technique for Group 1 patients

The surgery was performed under general anesthesia. Each patient was placed in the supine position with a sandbag under their posterior chest. An infraclavical incision followed the deltopectoral interval, and as the anterior axillary fold was crossed, the incision continued in a zigzag fashion, entering the axilla and arm to explore the brachial artery. When an injury was found in the first or second portion of the axillary artery or was combined with a clavicular fracture preoperatively, a transverse supraclavical incision was occasionally also made (Figure 1).

**Figure 3:**
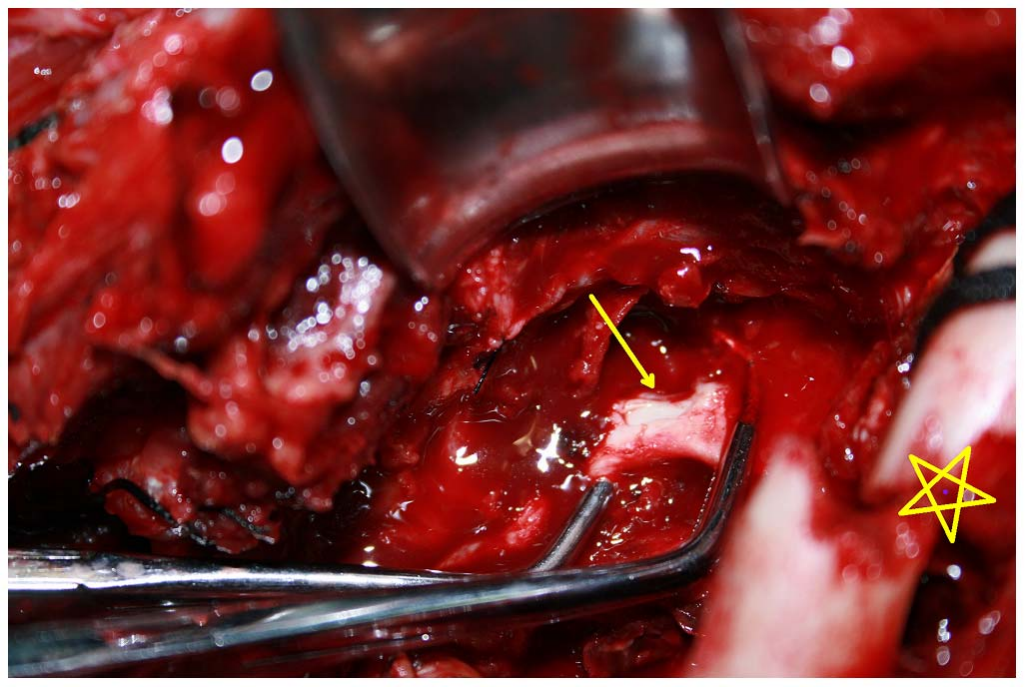
Patient no 6's vascular surgery. When the sac of pseudoaneurysm was removed, the injury of the axillary artery could be evaluated clearly. A 5-mm length of laceration was at the anterior wall of axillary artery in patient no. 6. (Arrow: the laceration of axillary artery; Pentacle: clavicle fracture).

**Figure 4:**
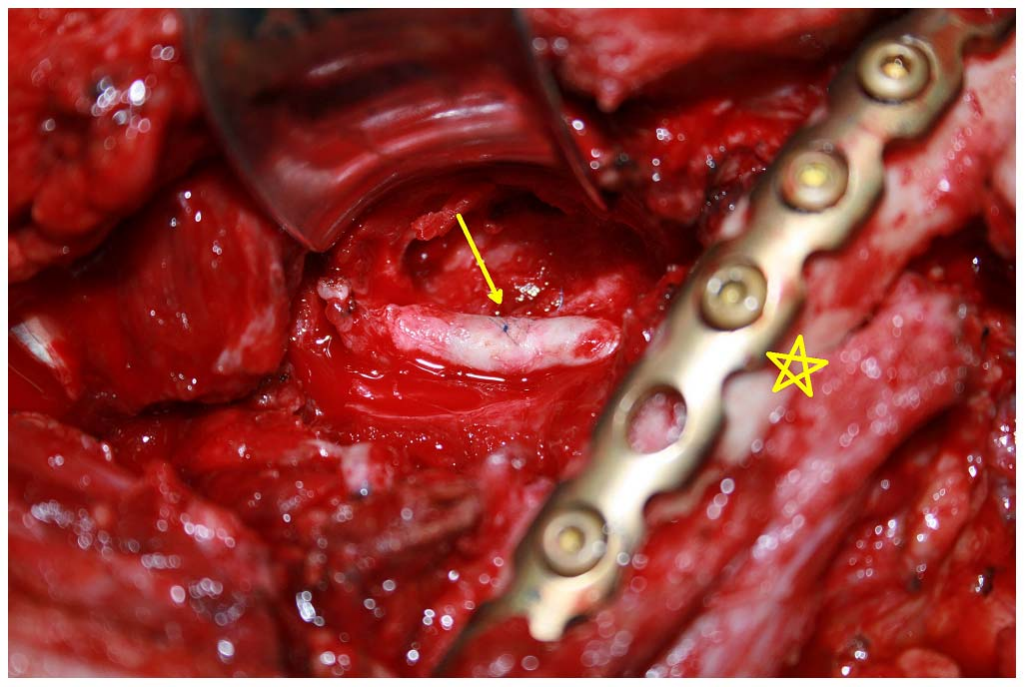
Patient no. 6's vascular surgery. The axillary artery wall was repaired directly, with clavicle fracture fixation simultaneously. (Arrow: the site of anastomosis; Pentacle: Clavicle fracture fixation).

The proximal and distal parts of the axillary artery, vein, and brachial plexus were identified and retracted with elastic tapes, and the soft tissues covering the pseudoaneurysm were detached. If an injury was found in the first or second portion of the axillary artery, it was beneficial to identify the distal part of subclavian artery through the transverse supraclavical incision to control the blood supply of the pseudoaneurysm. The proximal and distal parts of the axillary artery were clamped by atraumatic vessel clamps. The capsule of the pseudoaneurysm was opened directly. After the thrombus and accumulated blood were removed, the axillary artery injury was evaluated (Figure 3). Depending on the extent of the axillary artery injuries, either direct repair of the vessel wall or resection of the involved vessel followed by vessel anastomosis were performed (Figure 4). A vein graft was not required for any Group 1 patients (Table 4).

**Table 4:** Characteristics of axillary artery (AA*) injuries.

Case no.	Injury portion of axillary artery	Size of pseudoaneurysm** (cm)	Axillary artery injury in detail	Artery repair	Radial pulse	Follow-up Ultrasound	CTA
							
1	Third	20×15×8	Avulsion of posterior circumflex humeral artery from AA	Repaired AA wall	+	-	/
2	Second	8×6×5	10 mm laceration at lateral wall of AA	Repaired AA wall	+	-	/
3	Third	8×5×4	3 mm laceration at anterior wall of AA	Resected 4 mm involved vessel	+	-	/
				Anastomosed AA directly			
4	Third	15×8×8	8 mm laceration at medial wall of AA	Repaired AA wall	+	-	/
5	Second	5×4.5×4.5	5 mm laceration at lateral wall of AA	Resected 6 mm involved vessel.	+	-	/
				Anastomosed AA directly			
6	First	4.5×4×3	5 mm laceration at anterior wall of AA	Repaired AA wall	+	-	-
7	Second	3.5×3.5×3.5	8 mm laceration at posterior and medial wall of AA	Resected 10 mm involved vessel.	+	-	-
				Anastomosed AA directly			
8	Second	7.5×6×5	Laceration at inferior wall of AA	Repaired by a covered stent-graft	+	-	-
9	first	3.5×3.5×2.7	Laceration at posterior wall of AA	Repaired by a covered stent-graft	+	-	-

*: AA  =  axillary artery.

**: the size of pseudoaneurysm detected in the operation.

**Figure 5:**
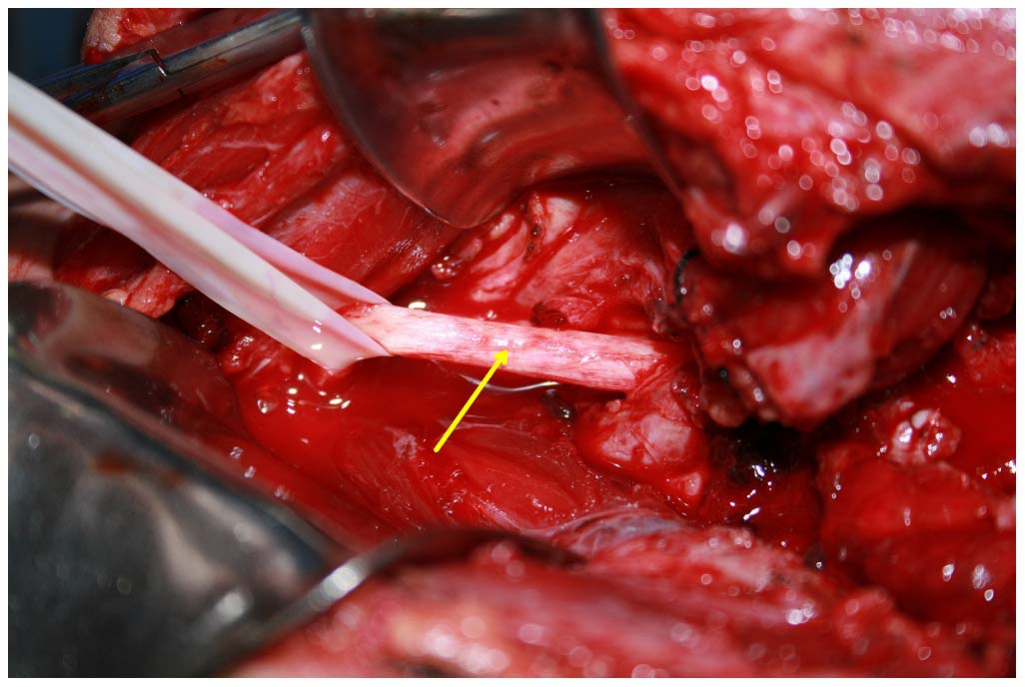
Patient no. 6's neurological surgery. The cords of the brachial plexus in patient no. 6 was explored. The lateral cord had a contusion but continuity was intact. (Arrow: The contusion of lateral cord).

After the pseudoaneurysm sac was removed, the brachial plexus was explored, and the method of repair was determined by the grade of the nerve injury. Only patient no. 4 sustained a partial severance injury of the medial cord. The continuities of brachial plexus in the other six patients were intact. Contusion of brachial plexus could be observed in all seven patients. Particularly in Patient no. 7, an obvious epineurium laceration on the medial cord was observed. If the brachial plexus had a contusion but its continuity was still maintained, microsurgical neurolysis was undertaken (Figure 5), as was seen in patient nos. 1–3, and 5–7. If the brachial plexus was found to have a rupture, nerve anastomosis was performed using an end-to-end suture method, as was seen in patient no. 4. Nerve grafting was not required for any Group 1 patients (Table 5).

**Table 5:** Characteristics of neurological deficits.

Case no.	Reason of nerve injury	Injured nerve	Neurological symptom	Nerve injury condition	Nerve surgery	Final recovery	Result of recovery motor/sensory*
1	Compression	Posterior cord	M3/S2	Contusion	Neurolysis	M5/S4	Good/Good
		Medial cord	M0/S0				
2	Compression	Lateral cord	M3/S2	Contusion	Neurolysis	M5/S4	Good/Good
		Posterior cord	M0/S2				
		Median cord	M0/S0				
3	Compression	Lateral cord	M0/S2	Contusion	Neurolysis	M5/S4	Good/Good
		Posterior cord	M2/S2				
		Medial cord	M0/S1	Partial rupture	Nerve repair	M3/S3+	Fair/Good
4	Traumatic and Compression	Lateral cord	M2/S3	Contusion	Neurolysis	M5/S4	Good/Good
		Posterior cord	M4/S3	Contusion	Neurolysis	M5/S4	Good/Good
		Medial cord	M1/S2				
5	Traumatic and Compression	Lateral cord	M0/S1	Contusion	Neurolysis	M5/S4	Good/Good
		Posterior cord	M0/S1				
		Medial cord	M3/S3				
6	Traumatic and Compression	Lateral cord	M0/S0	Contusion	Neurolysis	M5/S4	Good/Good
		Posterior cord	M3/S2				
		Medial cord	M0/S2	epineurium laceration		M3/S3+	Fair/Good
7	Compression	Lateral cord	M3/S3	Contusion	Neurolysis	M5/S4	Good/Good
		Posterior cord	M4/S4	Contusion		M5/S4	Good/Good
8	Traumatic and Compression	Lateral cord	M2/S2	Contusion+Neuroma Embedded in scar	Neurolysis	M4/S4	Good/Good
		Posterior cord	M0/S0		Nerve graft	M3/S3+	Fair/Good
		Lateral cord	M0/S0	Embedded in scar	Neurolysis	M3/S4	Fair/Good
9	Traumatic and Compression	Medial cord	M0/S0	Contusion	Neurolysis	M4/S3+	Good/Good
		Radial nerve	M3/S3	Contusion	Neurolysis	M5/S4	Good/Good
		Axillary N	M0/S0	Contusion	Neurolysis	M5/S4	Good/Good

*The result of neurological recovery is graded according to Birch's grading system [8].

Some patients with bone fractures also underwent open reduction internal fixation (ORIF). Following the operative procedures, drainage tubes were used to prevent hematoma formation.

Low-dose heparin 5000 IU was administered with a hypodermic syringe, and low molecular weight dextran was administered in an intravenous drip infusion every 12 hours for 5 days. The use of anticoagulants was discontinued after 5 days.

### Surgical Technique for Group 2 patients

The surgery was performed under general anesthesia with the patient placed in an identical fashion to that of Group 1 patients. An infraclavical incision followed the deltopectoral interval to an extent necessary for nerve exploration only; vascular surgery was not necessary in this subgroup. The brachial plexus was regularly explored, and the method of repair was determined by the grade of the nerve injury. In patient no. 9, the lateral cord of the brachial plexus was embedded by a thickened scar but continuity was still maintained; thus microsurgical neurolysis was performed. In patient no. 8, the posterior cord of the brachial plexus could not be separated from the solid hematoma mass, and an intraoperative electrophysiological examination showed complete injury of the posterior cord. The segment of the posterior cord that was embedded in the hard scar tissue was thus abandoned and 6 cm length cable nerve grafting was performed.

Because a stent was used for pseudoaneurysm before the surgery, the patients were asked to take aspirin 100 mg and clopidogrel 75 mg daily for at least 6 months. The day before surgery, the oral drugs were suspended and then reinitiated on postoperative day.

### Functional evaluations

Postoperatively, patients were reviewed daily until discharge, and then at approximately 1, 3, and 6 months postoperatively. Subsequently, the patients were advised to have follow-up examinations every 6 months. Follow-up examinations were as follows:

#### Pain evaluation

According to the VAS, pain symptoms, which included the initial pain and the pain at the time of admission, were assessed preoperatively. Then, the pain intensity was measured every 4 hours in the first postoperative week until discharge. The characteristics of the pain and its impact on daily life were also recorded. The intensity and characteristics of pain were further assessed during routine follow-up (Table 3).

#### Vascular evaluation

The radial artery pulse, skin temperature (monitored with a transistor thermoscope), color, swelling, and capillary blood refill of the relevant upper extremity were examined every 4 hours for 5 days postoperatively. Then, the radial artery pulse was palpated during routine follow-up, and CTA or ultrasound were used to further check the condition of the axillary artery unconventionally (Table 4).

#### Neurological evaluation

The neurological function was assessed according to the British Medical Research Council System. The grade of nerve function recovery was evaluated according to Birch R's grading system [8] (Table 5).

## Results

The follow-up duration ranged from 24 to 135 months (mean, 53.9 months).

### Pain evaluation (Table 3)

Before the vessel repair, whether by endovascular or operative treatment, the characteristics of pain were described by all patients as being constant, distending and pulsating in the axillary region and upper extremities. The degree of pain became more intense with time. On admission, all patients had somnipathy and required analgesics to control the pain.

For patient nos. 1–7, the degree of pain decreased markedly on postoperative day 1 and the type of pain changed to a stabbing pain. Analgesics were not required postoperatively. For patient nos. 8 and 9, pain decreased markedly after the endovascular treatment. The chief complaint changed thereafter to numbness and weakness prior to the nerve surgery. No patient complained of pain at last follow-up.

### Vascular evaluation

Eight patients had a good vascular condition after the vessel repair, whether by endovascular or operative treatment. Only one patient (no. 7) presented with a slowed capillary blood refill and the radial artery pulse could not be detected on postoperative day 3. An emergency DSA examination was performed and it showed that the repaired axillary artery was unobstructed but that the subclavian artery was occluded by a 3 cm long thrombus. Thrombolytic therapy and stent-grafting was then performed by a vascular surgeon, and the radial artery pulse was recovered. The patient required anticoagulant medicines for 6 months. All patients maintained a good radial artery pulse during the follow-up period (Table 4).

### Neurological evaluation

**Figure 6:**
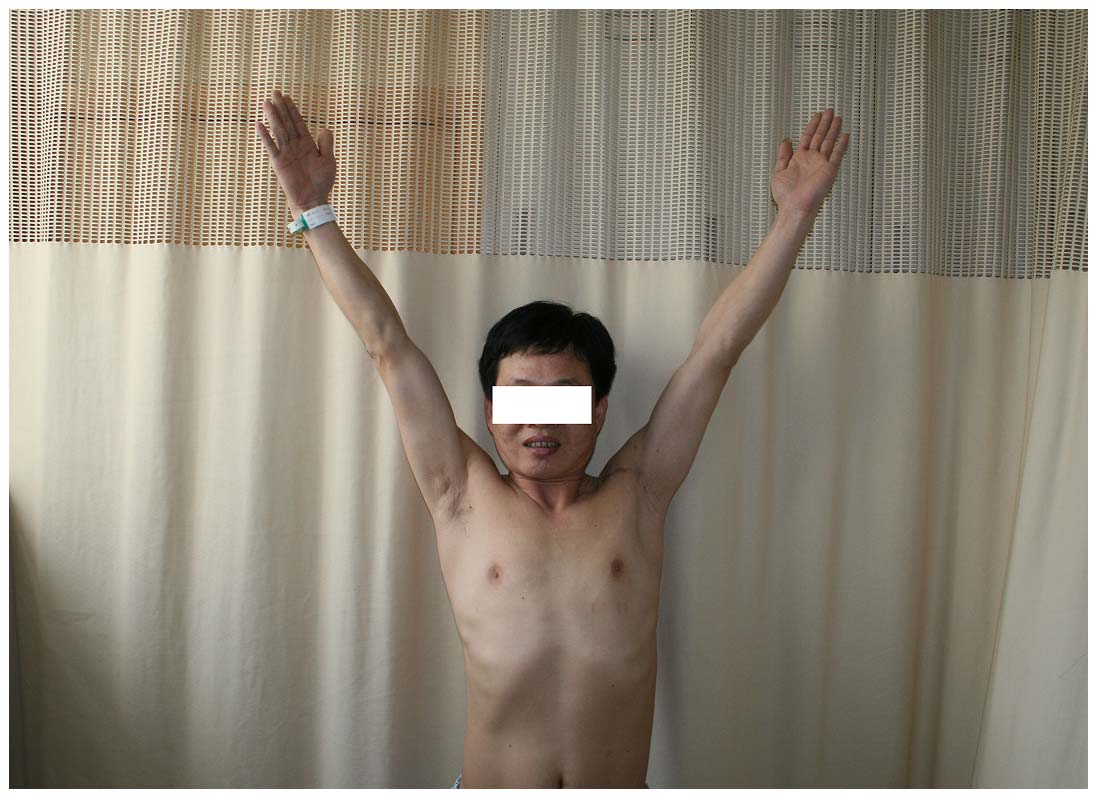
The postoperative results of patient no. 6 after 25 months.

**Figure 7:**
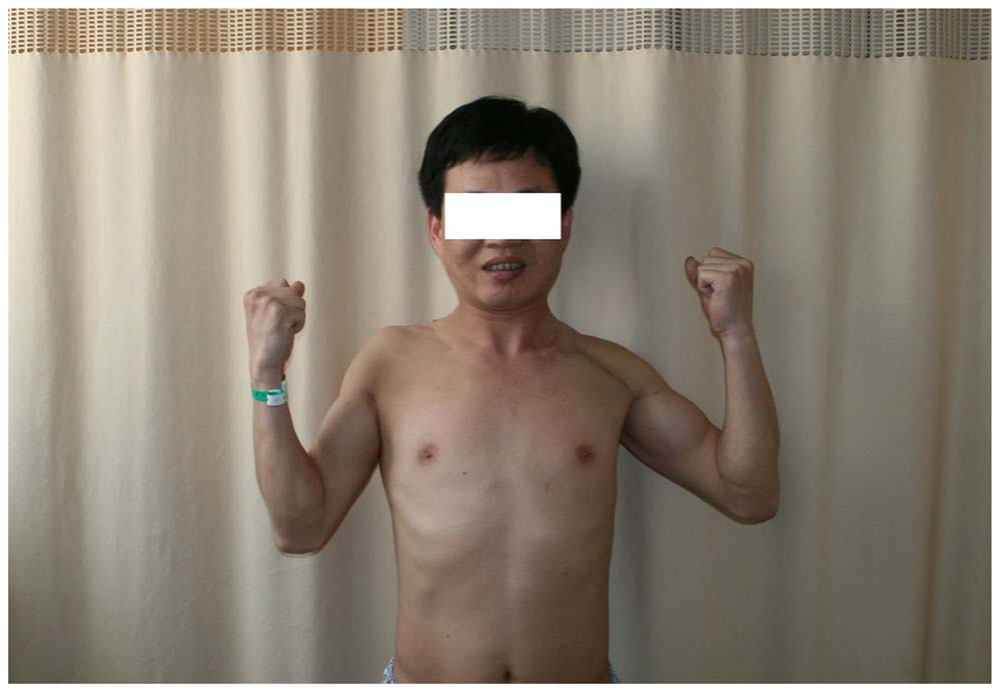
The postoperative results of patient no. 6 after 25 months.

The recovery of motor function was rated as good in five cases and fair in four cases, and the recovery of sensory function was good in all patients (Table 5) (Figure 6 and 7).

In the five patients with good results (patient nos. 1–3, 5 and 6), the continuities of brachial plexus were intact, and only neurolysis were undertaken. On the other hand, the four patients with fair motor function recovery (patient nos. 4, 7, 8 and 9) were those with definite nerve injuries: Patient no. 4 sustained a partial severance injury of the medial cord. In patient no. 7, an obvious epineurium laceration on the medial cord was observed. The patient nos. 8 and 9 with delayed nerve surgery, the brachial plexus was embedded by a thickened scar.

## Discussion

Traumatic pseudoaneurysms of the axillary artery are rarely encountered in civilian clinical practice, with case reports comprising the majority of the literature on this condition. Penetration trauma, such as stab wounds and gun missile wounds, is the most common cause of pseudoaneurysms [9]. In our series, four of nine patients (44.4%) had sustained a stab injury, another four patients (44.4%) had sustained a traffic accident injury, and the other one patient (11.1%) had sustained a fall accident injury. Thus, in contrast to previous reports [1], [2], [9], [10], [11], [12] we found that violent blunt trauma, e.g., from traffic accidents, was a notable cause of traumatic pseudoaneurysms of the axillary artery combined with brachial plexus injury.

### Clinical features and diagnosis of pseudoaneurysms

In general, traumatic axillary pseudoaneurysms arise from partial laceration of the axillary artery and subsequent bleeding from the lumen into the surrounding soft tissue. As the pseudoaneurysms expanded, pain or neurological deficit presents. However, although the surrounding bone and muscular girdle does provide considerable coverage and protection, it may obscure physical signs of vascular injury and hinder rapid operative exposure and vascular control. Some classic signs and symptoms of a pseudoaneurysm, such as expanding hematoma, continuous thrill, and a diminished radial artery pulse pressure, may be absent or unclear in this region. In our series of patients, only two (22.2%) had clear thrill masses and two (22.2%) had clear pulseless symptoms. For patient nos. 1–7, a diagnosis of pseudoaneurysm was missed during the initial evaluation in the primary hospitals.

Based on our findings, it would appear that constant pain, especially distending and pulsating pain, may be an important indicator of an injury of the axillary artery. All nine patients had constant, distending, and pulsating pain, which became more serious over time. After the vessel repair, whether by endovascular or operative treatment, the pain decreased markedly and the VAS pain score decreased from a preoperative value of 5.9 to 2.4. These changes in pain were characteristic and may be helpful for future diagnosis and confirmation of resolution of the condition.

If a pseudoaneurysm is suspected clinically, imaging modalities such as ultrasonic examination, CTA, MRA, or DSA may be useful to assist in diagnosis [13]. Compared with other imaging modalities, ultrasonic examination is cheap and non-invasive, and has the advantage of being able to be performed at the patient's bedside. All patients in our series underwent ultrasound examinations and achieved accurate diagnosis.

Digital Subtraction Angiography (DSA), when employed as the initial diagnostic modality for vascular injury, has the advantage of enabling the placement of a stent or occluding balloon as an endovascular treatment as required. However, conventional diagnostic DSA also has the disadvantage of being time-consuming and invasive, and may be associated with serious complications. CT angiography (CTA) is emerging as a novel modality to study arterial anatomy, with the additional advantages of being non-invasive and able to evaluate arterial trauma rapidly and reliably, reducing the delay before repair of the injury [14]. We employed CTA in three patients and achieved satisfactory results. It can provide valuable information for operative planning (Figure 2). Based on our observations, we now utilize ultrasound as a frontline diagnostic modality for such patients. And we recommend routine CTA use before surgery.

We did not use MRA in this study. Kapoor et al reported their experience and suggested that it may be useful as an alternative to CTA in patients who are contraindicated for CT contrast, e.g., those with an iodine allergy or reduced renal function [13].

### Clinical features and prognosis of brachial plexus injuries

Neurological compression may present as a complication in 10% to 30% of patients with a pseudoaneurysm [9]. A patient with a pseudoaneurysm could present with pain or signs of a neurological deficit several hours or many years after the initial injury [9], [15]. However, in patients with a traumatic axillary artery pseudoaneurysm combined with a brachial plexus injury, neurological compression can be closely related to the anatomic axilla and a host of neurovascular structures in this region.

Based on our findings, pain or signs of a neurological deficit may present more severely and dramatically when a pseudoaneurysm is present in the first or second portion of the axillary artery with a narrow space beneath the pectoralis minor, as was seen in patient nos. 2 and 5–9. Further, if the axillary artery injury occurred in the third portion, it would not compress the brachial plexus until the pseudoaneurysm became greatly enlarged in the axilla space over time, as was seen in patient nos. 1, 3, and 4. The range of intervals from trauma to neurological dysfunction was 0 to 9 days (mean, 2 days) in the former group of patients, compared with 0 to 60 days (mean, 24 days) in the latter. Further, the range of volumes of the pseudoaneurysms in the former group was 33 to 240 cm^3^ (mean, 116 cm^3^) compared to 160 to 2400 cm^3^ (mean, 1173 cm^3^) in the latter. Therefore, the injured portion of the axillary artery played an important role in the clinical symptoms of the traumatic axillary artery pseudoaneurysm combined with the brachial plexus injury.

Roganovic et al reported their experience in treating peripheral nerve lesions associated with missile-induced pseudoaneurysms, stating that the resulting nerve damage could be severe if surgery was delayed for more than 3 to 4 days after symptoms of neurological decline [9]. Jackson et al. also found that a compressed nerve left untreated for longer than 3 to 4 days was a critical factor in the development of severe damage resulting from a neurapraxic lesion, and immediate surgical decompression was recommended to avoid permanent nerve damage [15]. In our series, the duration of nerve compression before treatment ranged from 5 to 120 days (mean, 38.7 days). Five patients achieved a good result. Two patients (patient nos. 4 and 7) with definite medial cord involvement achieved reasonable motor recovery and good sensory recovery results. The other two patients (patient nos. 8 and 9) with delayed nerve surgery achieved good sensory recovery but fair motor recovery results.

Therefore, the key prognostic indicators of brachial plexus injury may be the nerve injury condition of brachial plexus and the treatment method, rather than the duration of the nerve compression.

### Endovascular treatment versus operative treatment

As endovascular repair techniques increase in popularity [6], [7], a growing number of surgeons are using vascular stent-grafts to treat pseudoaneurysms. However, for patients with traumatic axillary artery pseudoaneurysms combined with brachial plexus injuries, open surgical exploration for nerve injury is necessary, regardless of whether or not initial endovascular treatment was performed. Furthermore, our experience indicates that this surgery should be undertaken as soon as possible following any endovascular treatments.

An extended interval between these two treatments may lead to surgical challenges during the secondary procedure: firstly, use of anticoagulation after the endovascular treatment carries a risk of hemorrhage in the operative area; and secondly, the hematoma in the pseudoaneurysm may become organized and form a solid mass after endovascular therapy, which is much harder than the sac of a pseudoaneurysm, leading to great difficulties in freeing the brachial plexus from the scar tissue and providing complete decompression of the brachial plexus, as was seen with patient nos. 8 and 9.

Based on our experience with this cohort, open surgical exploration was the preferable treatment option to treat the axillary artery pseudoaneurysm combined with brachial plexus injury. Endovascular procedures can be viewed as a backup treatment option if the open procedure fails, as occurred with patient no. 7. The patient achieved reasonable nerve function recovery and good radial artery pulse during the follow-up period.

## Conclusions

In summary, both penetration and blunt trauma can cause an axillary artery pseudoaneurysm combined with brachial plexus injury. Violent blunt trauma has a high likelihood of causing immediate and serious neurological symptoms and fractures which could comprise the symptom of a pseudoaneurysm easily. Constant and expansive pain may be an important indicator in the diagnosis of a pseudoaneurysm. The characteristics of the symptoms are likely to correlate to the location of the injured axillary artery. Ultrasound examination and CTA are useful modalities to evaluate vascular injury and provide valuable information for operative planning. Open surgical exploration is an effective therapy, the results of which are linked to the nerve injury condition of brachial plexus.
